# Broccoli Leaves Attenuate Influenza A Virus Infection by Interfering With Hemagglutinin and Inhibiting Viral Attachment

**DOI:** 10.3389/fphar.2022.899181

**Published:** 2022-06-30

**Authors:** Won-Kyung Cho, Nam-Hui Yim, Myong-Min Lee, Chang-Hoon Han, Jin Yeul Ma

**Affiliations:** ^1^ Korean Medicine Application Center, Korea Institute of Oriental Medicine, Daegu, South Korea; ^2^ College of Veterinary Medicine, Jeju National University, Jeju, South Korea

**Keywords:** broccoli leaf ethanol extract, influenza A virus, hemagglutinin, virucidal effect, viral attachment

## Abstract

Broccoli (*Brassica oleracea* L. var. *Italica*) leaves are a byproduct of broccoli and could be used as a food source. The study aimed to evaluate the effect of broccoli leaves on influenza A virus (IAV) infection. We investigated the effect of ethanol extract of Broccoli leaves (EBL) on IAV infection using green fluorescent protein (GFP)–tagged Influenza A/PR/8/34 virus (PR8-GFP IAV). When EBL and PR8-GFP IAV were cotreated to RAW 264.7 cells, the fluorescence microscopy and fluorescence-activated cell sorting (FACS) analysis showed that EBL significantly reduced the levels of GFP expression by influenza viral infection dose-dependently. Immunofluorescence (IF) analysis confirmed that EBL decreased the expression of IAV proteins. EBL exhibited a strong inhibitory effect of IAV binding on the cells and moderate virucidal impact. Consistently, EBL potently suppressed the hemagglutination by IAV infection. These results indicate that EBL prevents IAV attachment *via* the inhibition of HA upon viral infection. Finally, EBL as an HA inhibitor of IAV could be used as the natural antiviral source to protect against influenza viral infection.

## Introduction

Influenza viruses belong to the family Orthomyxoviridae and have segmented negative-strand RNA (Bouvier and Palese, 2008). Influenza A virus (IAV) mainly causes respiratory infections, muscle and joint pain, and pneumonia and seriously affects people, causing even death in high-risk groups. Every year seasonal influenza viruses infect 5–15% of the global human population, and it caused approximately 290,000 to 650,000 deaths in 2017 (Clayville, 2011; Paget et al., 2019). IAV is responsible for epidemics and pandemics due to new variants originating from frequent antigenic drift and antigen point mutation (Dunning et al., 2020). The genetic re-assortments from human and avian and/or swine influenza viruses result in significant mutations in antigenic sites of the influenza virus (Shao et al., 2017; Petrova and Russell, 2018). This antigenic drift is the main reason for new variants containing novel hemagglutinin (HA) and/or neuraminidase (NA) of the influenza virus (Shao et al., 2017; Petrova and Russell, 2018). However, it is impossible to predict the next new antigen drift of IAV to prepare the perfect vaccine development. In this respect, it is critical to develop antiviral agents to control IAV-related damages (Berlanda Scorza et al., 2016). Antiviral inhibitors used in clinical practice include the M2 protein inhibitors such as amantadine and rimantadine and neuraminidase inhibitors such as oseltamivir, zanamivir, peramivir, and baloxavir. But, there are lot of reports on the side effect and drug resistance to these inhibitors (Hussain et al., 2017). It is inevitable to discover a new natural antiviral agent against influenza viral infection. Natural products such as herbs, foods, and minerals contain various ingredients, which could exert the synergy effect on drug efficacy and alleviate the side effect of synthetic compounds.

Broccoli (*Brassica oleracea* L. var. *Italica*), which is one of the most consumed vegetables, contains high levels of nitrogen-sulfur derivatives such as glucosinolates, isothiocyanates, phenolic compounds, including chlorogenic acids and flavonols*.* Several reports demonstrated Broccoli extract could prevent cancer (Elkashty et al., 2018; Elkashty and Tran, 2020), cardiovascular diseases (Rodriguez-Cantu et al., 2011; Zandani et al., 2021), diabetes (Xu et al., 2016; Crunkhorn 2017), and obesity (Xu et al., 2018). Most research on broccoli was restricted to an edible floret, sprouts, or its main compound, sulforaphane (SFN). Broccoli leaf was not used as the main food source and was discarded as waste as a byproduct. A recent report showed that broccoli leaves ameliorated amyloid-beta (Abeta)1-42-induced learning and memory impairment (Park et al., 2016). Le T.N. et al. performed a comparative study to examine polyphenol and bioactive compound contents in broccoli’s edible and non-edible parts. Broccoli leaf exhibited strong antioxidant activity with high polyphenolic contents and exerted an antibacterial effect, comparable to that by the floret (Le et al., 2020). However, the antiviral effect of broccoli leaf extract was not reported. In the present study, we first demonstrate the anti-influenza viral efficacy of broccoli leaves through direct inhibition of hemagglutinin.

## Results

### The Cytotoxicity of Broccoli Leaves Ethanol Extract

**FIGURE 1:**
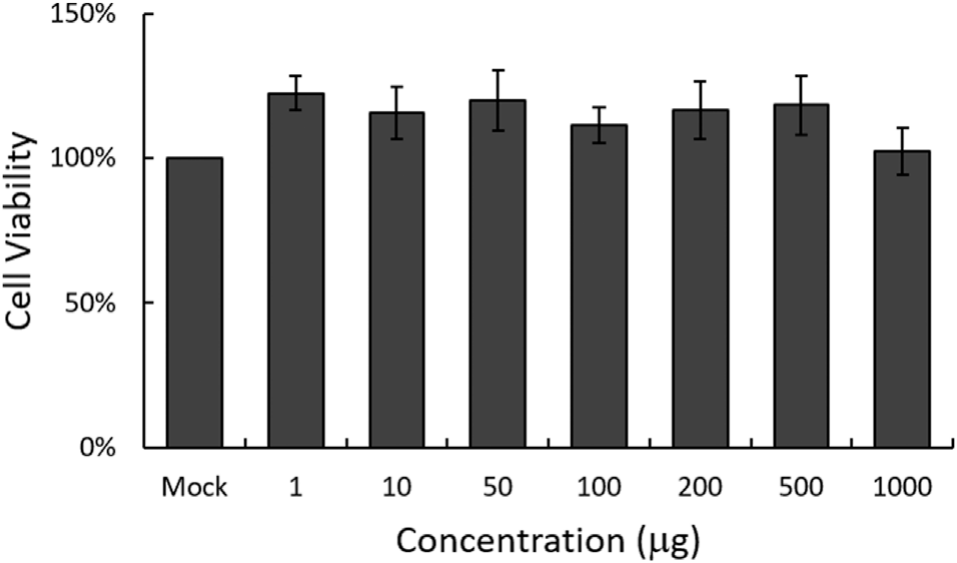
Determination of the cytotoxicity of EBL in RAW 264.7 cells. EBL was added to the cells with indicated concentrations for 24 h at 37°C. Cell viability was assessed by CCK-8 assay. The data represent the mean ± SD based on three replicates in three different experiments.

To examine the cytotoxicity of EBL on RAW 264.7 cells, we used CCK-8 assay. As shown in Figure 1, EBL did not show significant cellular toxicity at the concentration of 1000 μg/ml.

### Antiviral Effect of Broccoli Leaves Extract on Influenza A Virus

To evaluate the antiviral effect of EBL against influenza A virus *in vitro*, the RAW 264.7 cells were cotreated by GFP-tagged Influenza A/PR8/34 (PR8-GFP) virus and 10, 100, or 200 μg/ml of EBL. The inhibitory effect of EBL on viral infection was measured through the levels of GFP expression by viral replication.

**FIGURE 2:**
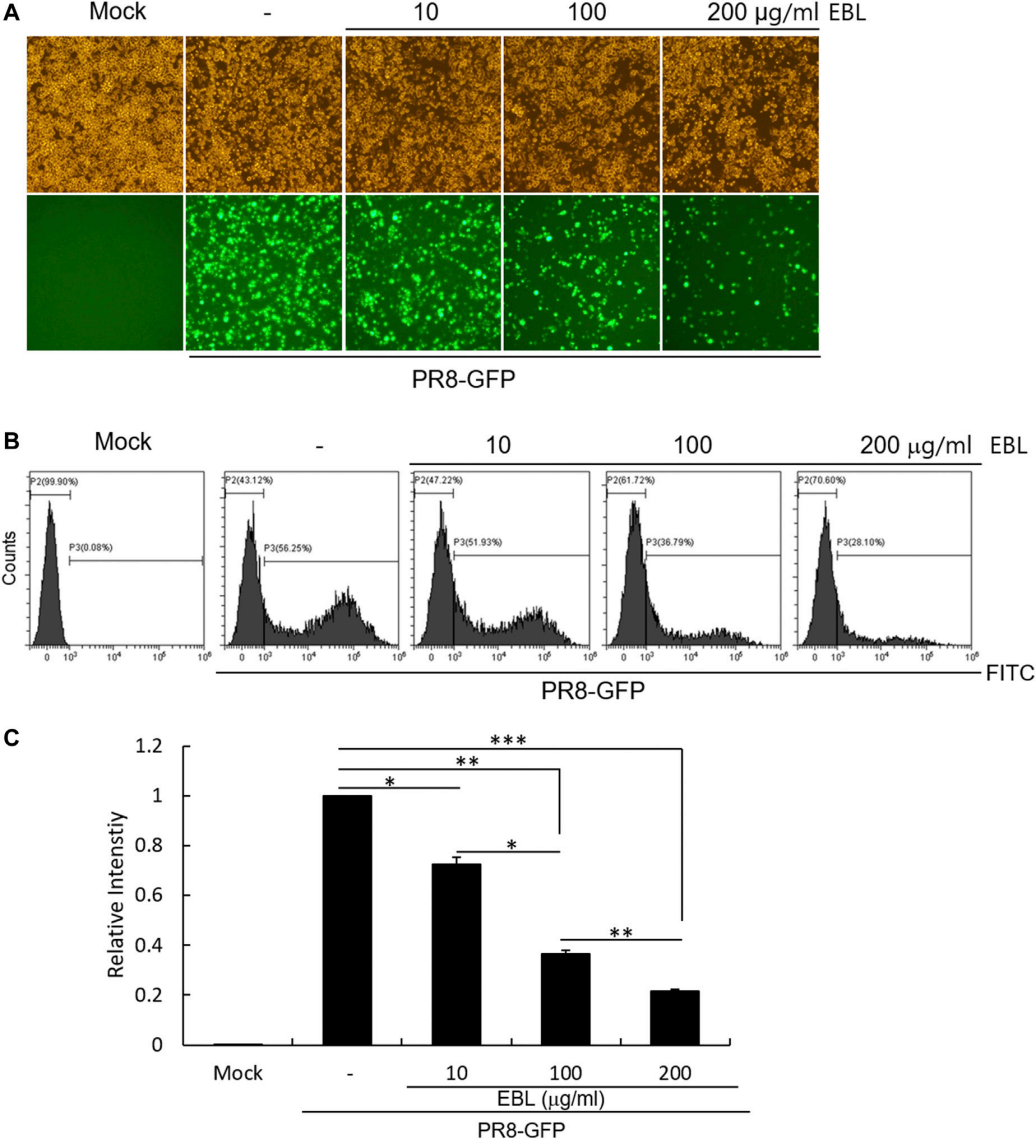
EBL exerted the inhibitory effect on influenza A/PR/8/34-GFP viral infection in a dose-dependent manner. EBL (0, 10, 100, or 200 μg/ml) was mixed with PR8-GFP IAV for 1 h at 4°C. The mixtures were added to RAW 264.7 cells for 2 h at 37°C. After washing with PBS, the cells were further incubated for 24 h. **(A)** Brightfield and fluorescence images were captured with the fluorescent microscope at a ×200 magnification. **(B)** After fixing with 4% paraformaldehyde and resuspended in PBS, the cells were analyzed for GFP expression by FACS. **(C)** Levels of GFP expression were depicted as relative intensities compared to those of the control PR8-GFP IAV. The data represent the mean ± SD based on three independent experiments. Statistical significance was assessed *via* an unpaired Student T-test. ***p < 0.0001, **p < 0.001, and *p < 0.005.

When the cells were infected with PR8-GFP IAV in the absence of EBL, the expression of GFP was detected by fluorescent microscopy. But, EBL significantly reduced the levels of GFP expression dose-dependently (Figure 2A). Next, we confirmed the inhibitory effect of EBL on PR8-GFP IAV infection using FACS analysis. The cells were uninfected, infected with PR8-GFP virus only, or with PR8-GFP and EBL extract mixtures at the indicated concentrations. At 24 h post infection, the cells were fixed with 4% paraformaldehyde and subjected to count GFP-expressing cells by measuring of the FITC level (Figure 2B). The levels of GFP expression in each group were compared with those of the group infected with the PR8-GFP virus, and the values of each group were graphed as relative intensities (Figure 2C). Consistent with Figure 2A, EBL remarkably repressed GFP expression by viral infection. These results indicate EBL has a strong inhibitory effect on PR8-GFP influenza viral infection.

### Broccoli Leaves Extract Reduces the Expression of Influenza Virus Proteins

**FIGURE 3:**
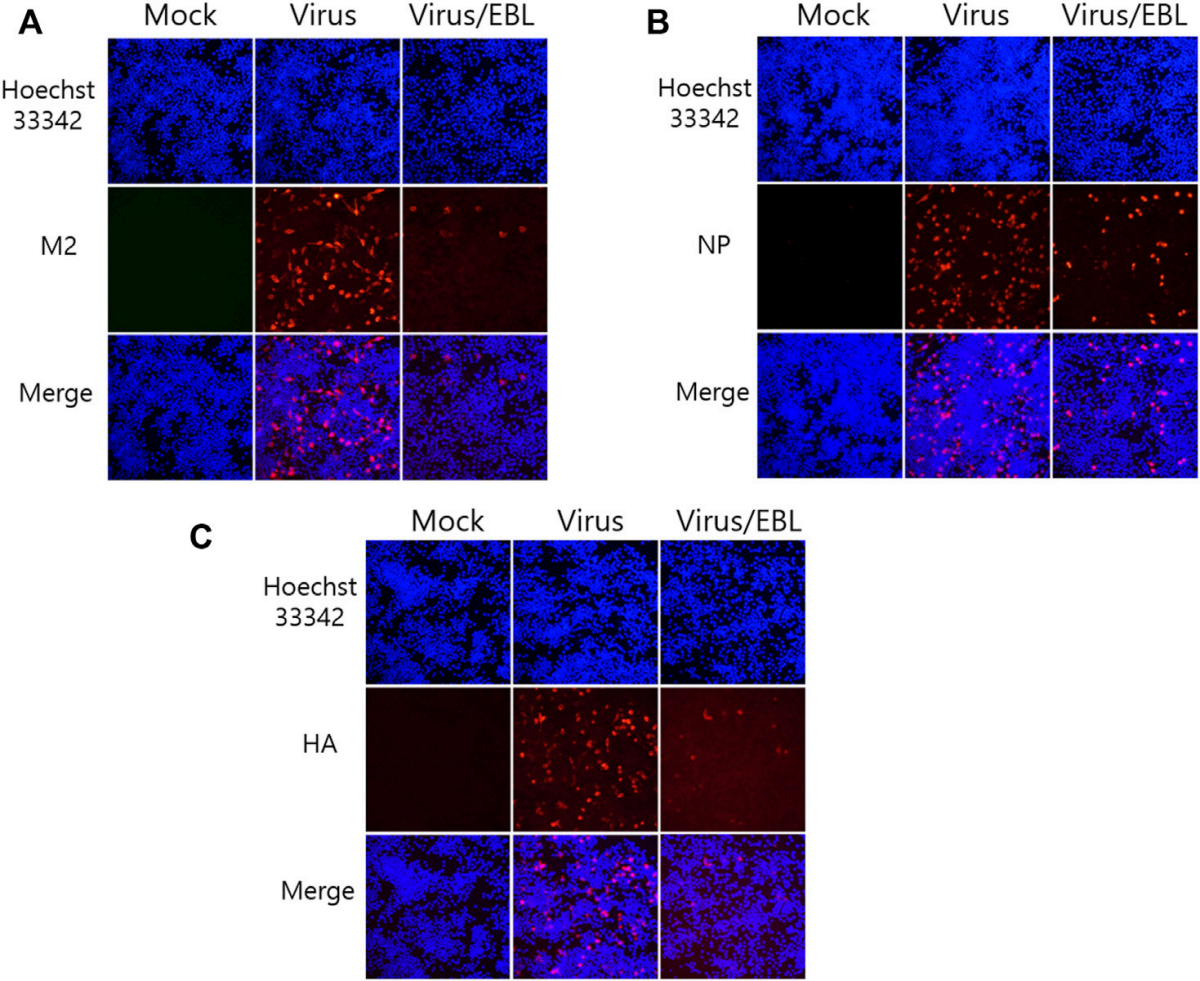
Repressive effect of EBL on influenza A/PR/8/34-GFP viral protein expression. EBL at 200 μg/ml or medium (mock) and PR8-GFP IAV were cotreated to the RAW 264.7 cells. At 24 h post infection, the cells were fixed with paraformaldehyde, stained with antibodies against **(A)** M2, **(B)** NP, **(C)** HA (red), and with Hoechst 33342 for nuclei (Blue). The co-localization of viral proteins and nuclei was observed under a fluorescent microscope.

Since EBL exhibited a strong inhibitory effect against IAV infection, we investigated whether EBL affects viral protein expression. EBL and PR8-GFP IAV were co-incubated at 4°C for 1 h before infection to RAW 264.7 cells. The mixtures were incubated for 24 h at 37°C until viral proteins were expressed. After fixing, the cells were stained with antibodies against IAV proteins, followed by staining with Hoechst 33342. The expression levels of IAV proteins were examined by immunofluorescence analysis. As shown in Figure 3, EBL reduced M2, NP, and HA proteins. Especially, M2 and HA proteins of IAV were significantly decreased by EBL (Figures 3A,C).

### The Effect of the Broccoli Leaves Extract on Viral Attachment, Entry, Virucidal Stages

**FIGURE 4:**
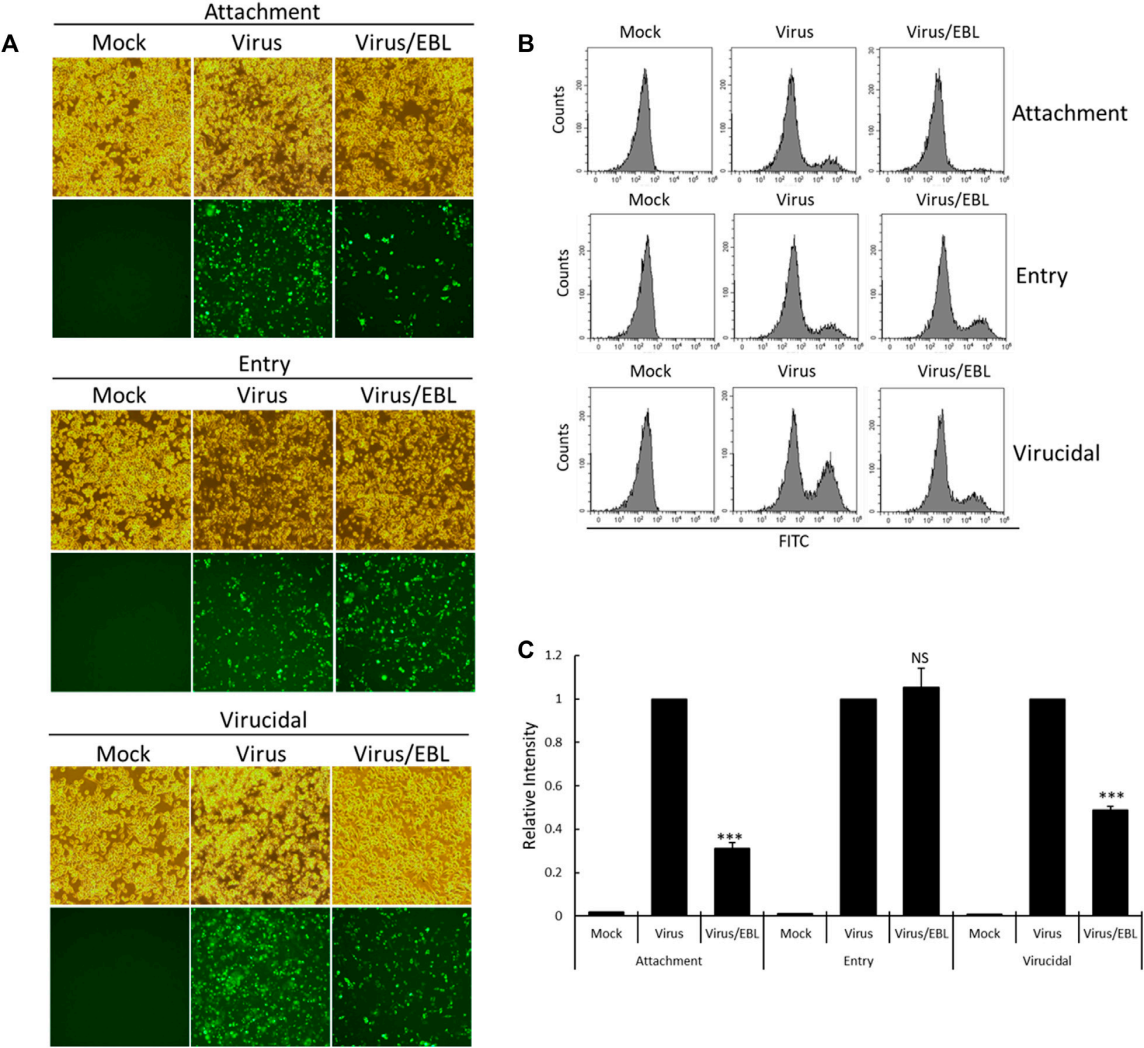
EBL suppresses viral attachment and exerts a virucidal effect. **(A–C)** For the attachment stage, RAW 264.7 cells were cotreated with PR8-GFP IAV and 200 μg/ml EBL at 4°C for 30 min. After washing, the cells were incubated at 37°C for 24 h. For the entry stage, the cells were infected with PR8-GFP IAV at 4°C for 30 min followed by the treatment of EBL at 37°C for 30 min. After washing, the cells were incubated at 37°C for 24 h. For the virucidal stage, PR8-GFP IAV and EBL were incubated at 4°C for 30 min. The mixtures were added to the cells and incubated at 37°C for 30 min. After washing, the cells were incubated at 37°C for 24 h. **(A)** Brightfield and fluorescence images of cells were captured under fluorescence microscopy with ×200 magnification. **(B,C)** RAW 264.7 cells were fixed and analyzed by FACS. The GFP-expressing cells were detected with FITC, and the values of relative intensities were calculated from comparison with PR8-GFP IAV. The data represent the mean ± SD value based on three independent experiments. Statistical significance was assessed *via* an unpaired Student t-test. ***p < 0.0005, NS: No significance.

Since EBL showed the strong anti–IAV activity in cotreatment, we performed the time-of addition assay to investigate what stages are inhibited by EBL upon IAV infection. To carry out that, we examined the effect of EBL on viral attachment, entry, or direct killing (virucidal) steps in IAV infection. To examine the inhibitory effect of EBL on the viral attachment stage, medium (mock), PR8-GFP or PR8-GFP and EBL mixture were added to the RAW 264.7 cells and incubated for 30 min at 4°C. After washing with PBS, the cells were incubated for 24 h at 37°C. To check the effect of EBL on viral entry into the cells, the RAW 264.7 cells were infected with medium or PR8-GFP at 4°C for 30 min before the addition of EBL. After the removal of the virus with washing, the medium (mock) or EBL was added to the cells at 37°C for 30 min. After washing, the cells were incubated at 37°C for 24 h. To examine whether EBL could kill the virus before infection onto the cells, medium (mock), PR8-GFP IAV, or PR8-GFP and EBL mixture were incubated at 4°C for 30 min. Each mixture was cotreated to the RAW 264.7 cells at 37°C for 30 min. After washing, the cells were incubated at 37°C for 24 h. As shown in Figure 4, in the presence of EBL, virus attachment was potently repressed, but entry was not. In addition, EBL showed moderate virucidal efficacy. These findings suggest that EBL has a substantial antiviral efficacy against influenza virus *via* the repression of viral attachment to the cells and virucidal effect upon infection.

### The Effect of the Broccoli Leaves Extract on Hemagglutination

**FIGURE 5:**
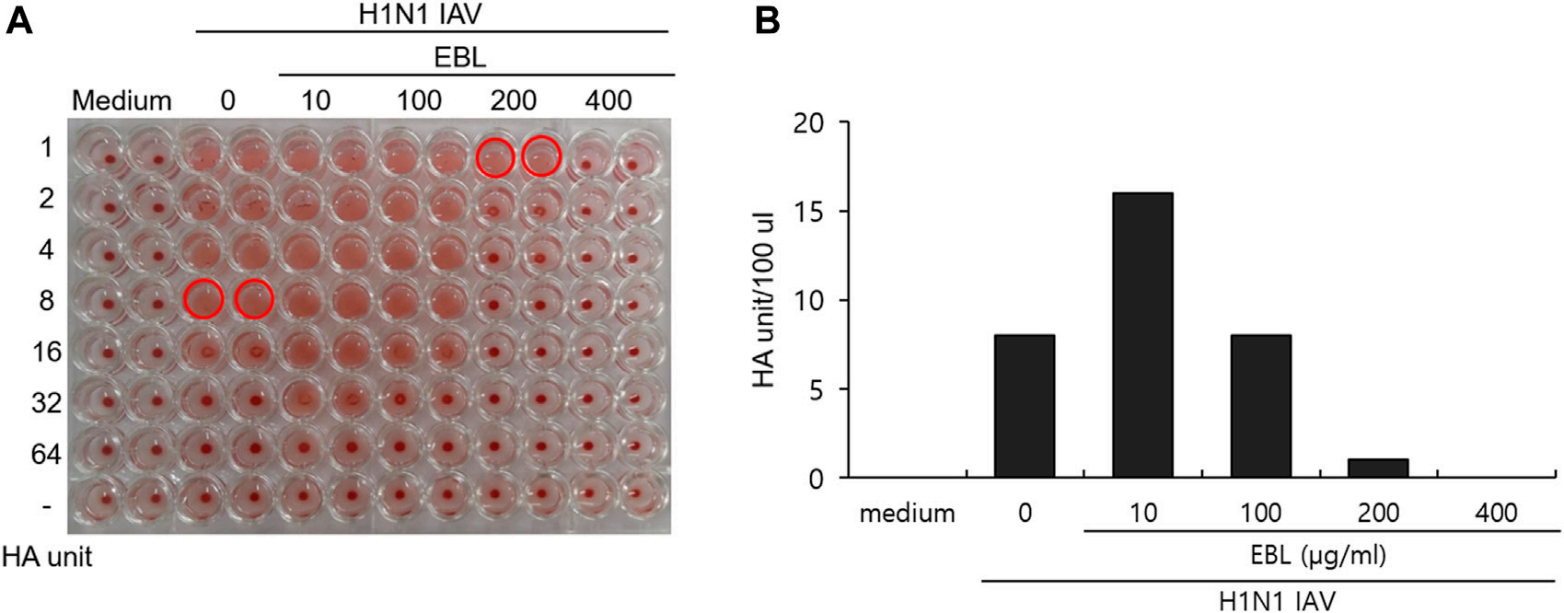
Inhibitory effect of EBL on hemagglutination by IAV infection. **(A,B)** EBL at concentrations of 10, 100, 200, or 400 μg/ml or medium was mixed with H1N1 IAV for 1 h at 4°C. The mixture was used to cotreat onto the RAW 264.7 cells. At 24 h post incubation, the supernatants of cells were serially diluted and mixed with RBC cells to examine the effect of EBL on hemagglutination by IAV infection.

HA of influenza virus is a critical protein for viral binding to the cells upon infection and is known to induce hemagglutination of RBCs. From the result of the strong inhibitory effect of EBL on the attachment of IAV onto the cells (Figure 4), we investigated whether EBL has an inhibitory effect on HA of IVA. The RAW 264.7 cells were infected with H1N1 IAV in the presence of EBL. After 24 h incubation, the supernatants of medium (mock), H1N1 virus, and the mixture of H1N1 and EBL were harvested, serially diluted, and mixed with chicken red blood cells for 1 h. As shown in Figure 5, EBL at 200 and 400 μg/ml showed a strong inhibitory effect on HA of H1N1 IAV. HA units of control H1N1 virus were 8 units, but in the presence of EBL at 200 μg/ml, the HA unit of viruses was 1 unit, and at 400 μg/ml, hemagglutination was not detected as a mock control. These results indicate that EBL strongly inhibits hemagglutination at 200 or 400 μg/ml.

### The Effect of the Broccoli Leaves Extract on Neuraminidase Activity

**FIGURE 6:**
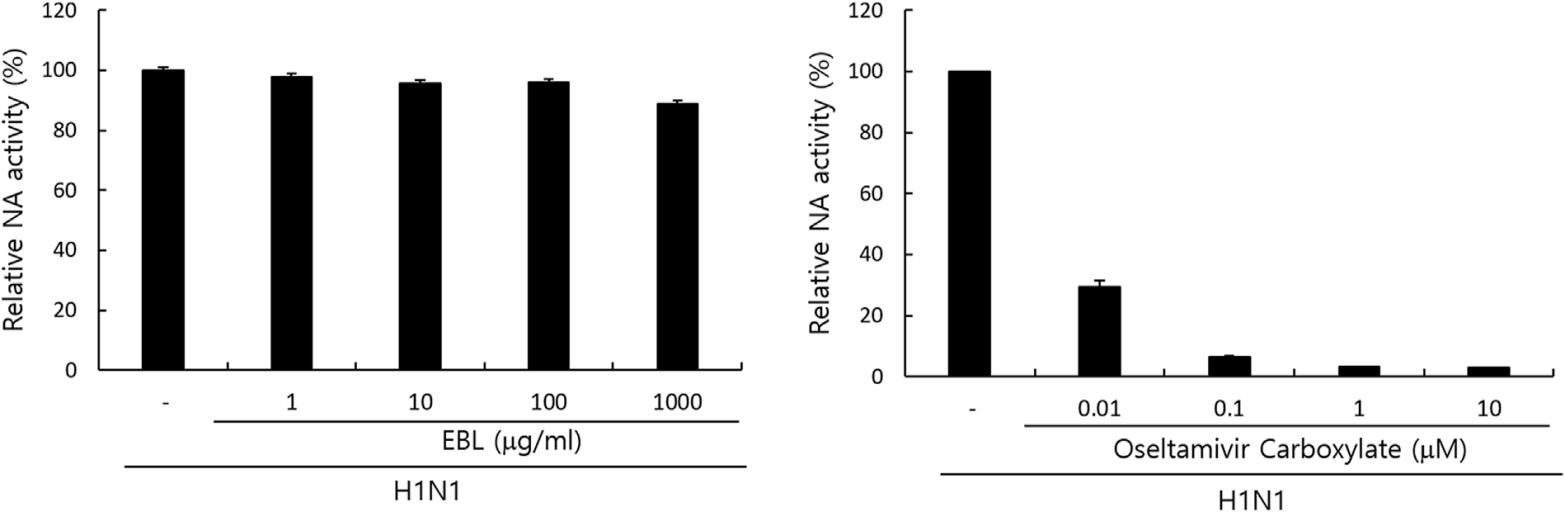
Effect of EBL on neuraminidase activity. EBL or oseltamivir carboxylate at indicated concentrations was mixed with H1N1 IAV in 96-well black plates. Each mixture was added with 200 µM NA-Fluor Substrate, incubated for 1 h for 37°C, and terminated by NA-Fluor stop solution. The NA activities were measured from the values in a fluorescent spectrometer using an excitation wavelength of 365 nm and an emission wavelength of 445 nm. The data represent the mean ± SD based on three independent experiments. Statistical significance was assessed *via* an unpaired Student’s T-test. ***p < 0.0005, **p < 0.001, and *p < 0.005.

Since neuraminidase activity plays a role in the release of IAV progeny from the cells, we examined whether EBL could affect NA activity. Oseltamivir carboxylate repressed NA activity up to more than 70% from a concentration of 0.01 μM, but EBL did not inhibit NA activity (Figure 6). This result suggests EBL does not inhibit IAV progeny release from the cells at the late stage upon IAV infection.

### The Effect of Sulforaphane on Influenza A Virus Infection

**FIGURE 7:**
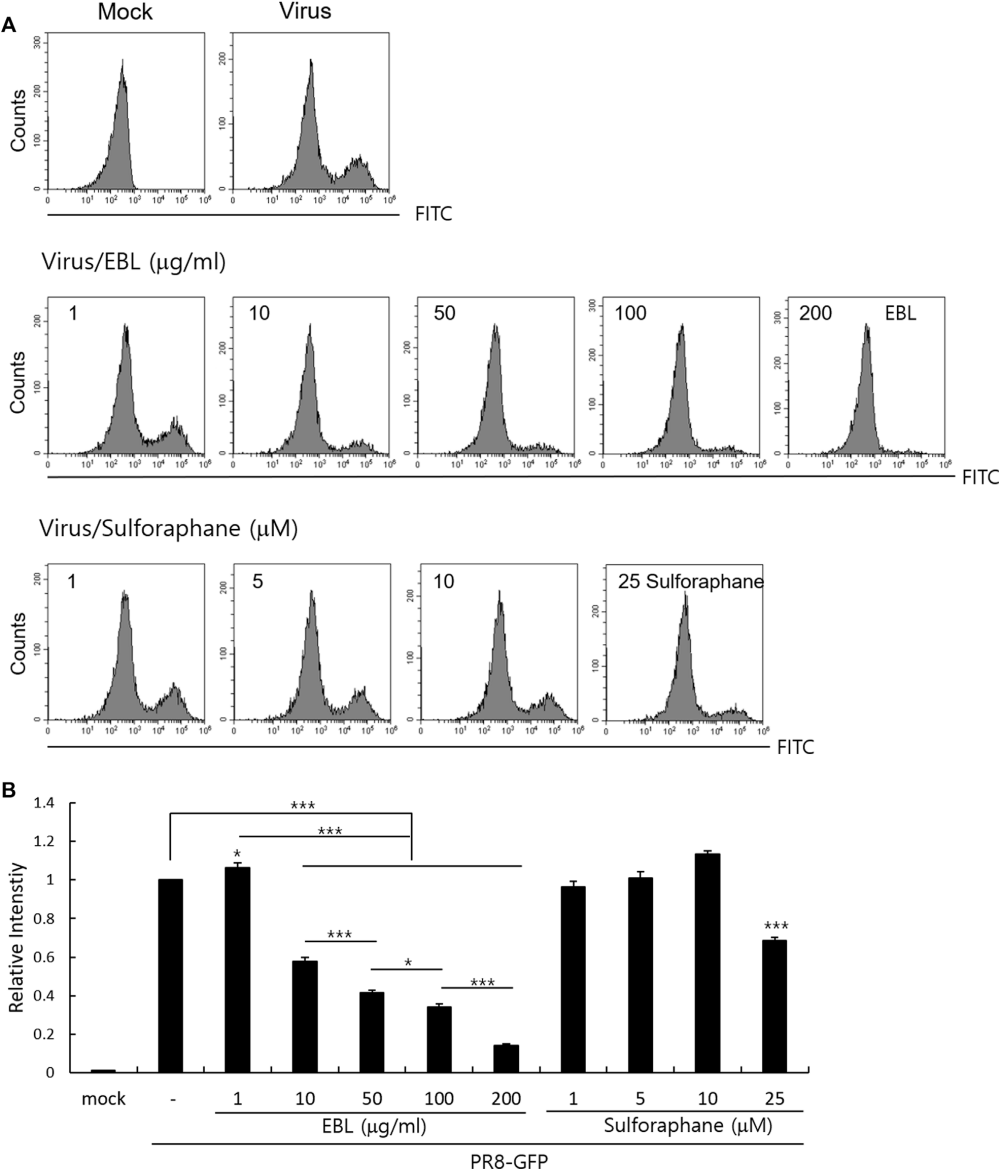
Effect of sulforaphane on IAV infection. **(A,B)** EBL or sulforaphane at indicated concentrations was mixed with PR8-GFP IAV and cotreated to RAW 264. 7 cells. The cells were washed with PBS at 24 h post infection, fixed with 4% paraformaldehyde, and resuspended in PBS for FACS analysis. The levels of GFP expression were depicted as relative intensities compared to those of the control PR8-GFP IAV. The data represent the mean ± SD based on three independent experiments. Statistical significance was assessed *via* an unpaired Student T-test. ***p < 0.005, **p < 0.01, and *p < 0.05.

Because sulforaphane (SFN), a main compound of broccoli, was detected in EBL in the UPLC analysis (Supplementary Figure S1), we investigated whether sulforaphane could inhibit IAV infection in the cotreatment trial. We compared the antiviral effects of EBL (1, 10, 50, 100, or 200 μg/ml) and SFN (1, 5, 10, and 25 µM) using PR8-GFP IAV expression and FACS analysis as described. Figure 7 represents that SFN moderately suppresses IAV at 25 μM. In addition, when we checked the effect of glucoraphanin (GRN) on IAV infection, we found that GRN did not affect IAV infection at all (data not shown). These results suggest that a significant anti-IAV impact of EBL is not related to SFN or GRN.

## Discussion

In this study, we demonstrated broccoli leaves extract directly inhibits influenza viral infection by preventing viral binding to cells at an early stage. We examined the effect of EBL on IAV infection using PR8-GFP IAV, which expresses GFP with IAV gene expression. The fluorescence microscopy and FACS analysis showed that EBL dose-dependently inhibits IAV infection. Consistently, immunofluorescence staining showed EBL repressed IAV protein expression. When we performed the time-of addition experiment to investigate in what stage EBL inhibits IAV infection, we found that EBL strongly inhibited the viral attachment stage, not the entry stage. IAV infection begins by the binding of HA of IAV and sialic acid–linked glycoprotein receptors on the target cells in the early stage of IAV infection. Several natural products such as cranberry extract (Luganini et al., 2018), *Jatropha curcas* (Patil et al., 2013), *Isatis indigotica* (Yang et al., 2012), *Camellia sinensis* (Song et al., 2005), and *Eupatorium perfoliatum* (Derksen et al., 2016) were shown to have an antiviral effect against IAV infection by modulating HA activity and viral attachment. We confirmed that EBL dose-dependently suppresses HA through hemagglutination inhibition assay. EBL at 200 μg/ml exerted 8-fold lower hemagglutination, and at 400 μg/ml of EBL, it completely blocked hemagglutination. These results support that EBL represses the viral attachment to the cell membrane *via* HA inhibition during IAV infection. Some compounds with triterpene and pentacyclic triterpene structure in natural products have shown antiviral effects by the binding to HA on the viral envelope, disrupting the interaction of HA with the sialic receptor and thus the attachment of viruses to the cells (Zu et al., 2012; Haruyama and Nagata, 2013; Li et al., 2013; Yu et al., 2014).

A recent report showed that SFN-rich broccoli sprouts attenuate IAV infection *via* activating T cells and NK cells (granzyme B) reduced by IAV infection (Muller et al., 2016). Since SFN is a major component of broccoli and detected in EBL using UPLC analysis, we also investigated the effect of SFN on IAV infection. Although SFN has been reported to contain antiviral efficacy against various viruses such as human immunodeficiency virus (HIV) (Furuya et al., 2016), hepatitis C virus (HCV) (Yu et al., 2016), Herpes simplex virus (HSV-1) (Schachtele et al., 2012), and Epstein–Barr virus (Wu et al., 2013), the anti-IAV effect of SFN was not significant in the cotreatment trial. EBL exhibited a moderate virucidal effect, not affecting NA activity. There are many research results on natural products with virucidal effects on IAV (Makau et al., 2013; Watanabe et al., 2014; Luganini et al. 2018; Li et al., 2019; Cho and Ma, 2022; Mohamed et al., 2022). Cranberry extract interacted with the ectodomain of viral HA glycoprotein, thus resulting in the interference with HA function and a consequent loss of infectivity of IV particles (Luganini et al., 2018). It needs to investigate which component in EBL binds to HA or directly kills the virus to exert a virucidal effect. Taken together, EBL inhibits influenza viral attachment to the cells at an early stage *via* the inhibition of hemagglutinin and virucidal effect at IAV infection. Despite no effect on NA activity, EBL exerted a potent anti-IAV impact. In this regard, EBL could be used for cotreatment with NA inhibitors such as oseltamivir or zanamivir to protect against IAV infection. Although further studies are needed to confirm the anti-IAV effect of EBL using *in vivo* experiments and identify the crucial constituents with potent anti-IAV effect in EBL and the related underlying mechanism, EBL has a significant anti-IAV effect and could be used as the natural antiviral agent to prevent IAV infection.

## Materials and Methods

### Cells and Viruses

RAW 264.7 cells (Mouse Leukemic Monocyte Macrophage cell line; ATCC TIB-71) were cultured in Le Roswell Park Memorial Institute medium (RPMI) (Hyclone, Logan, UT) with 10% fetal bovine serum and 100 U/ml of penicillin and streptomycin at 37°C with 5% CO_2._ Green Fluorescent Protein (GFP)–tagged Influenza A/PR8/34 (PR8-GFP) and A/PR8/34 (H1N1) viruses were kindly gifted from Prof. Jong-Soo Lee (Chungnam National University, Daejeon, South Korea) and propagated in the allantoic fluid of a 10-day old chicken embryo as described previously (Cho et al., 2015; Choi et al., 2016; Moon et al., 2017). All virus-related experiments were performed at the BL2 (Biosafety level 2) level.

### Preparation of Broccoli Leaves Ethanol Extract and Ultra-Performance Liquid Chromatography Analysis

Broccoli (*Brassica oleracea* L. var. *Italica*) leaves ethanol extract was provided by Professor Chang-Hoon Han (College of Veterinary Medicine, Jeju National University). The preparation of ethanol extract of broccoli (EBL) and ultra-performance liquid chromatography (UPLC) analysis to detect sulforaphane (SFN) and glucoraphanin (GRN) was described in the previous report (Ranaweera et al., 2021). Briefly, small pieces of broccoli leaves in 10 volume water were treated with pulsed electric field (PEF) using 5 kW PEF at 7 kJ of total energy for 5 s (out voltage 60%, pulsed width 25 µs, frequency 100 Hz). The suspension mixed with 10 volumes of ethanol was extracted and stored at −80°C until use. Chromatographic analysis was performed using an analytical AQUITY UPLC BEH C18 column (2.1 × 100 mm, 1.7 μm : Waters Corporation, MA, United States), the mobile phase consisted of water containing 0.1% formic acid (solvent A) and acetonitrile containing 0.1% formic acid (solvent B). The gradient elution system was conducted as follow: 0.0 min, 2% B; 5 min, 7% B; 10 min, 20% B. SFN was detected at 205 nm and GRN was detected at 220 nm. The dried EBL was dissolved in 50% DMSO and used for antiviral experiments.

### Detection of Cytotoxicity

The CCK-8 assay was used to evaluate the cytotoxicity of EBL on the cells, according to the manufacturer’s recommendation (Dojindo, Rockville, MD, United States). Briefly, RAW 264.7 cells were seeded in 96-well plates (1 × 10^5^ cells/well) and incubated with EBL at concentrations from 1 to 1000 μg/ml. After 24 h, 10 μl of the reagent was added to the plate, and then the absorbance at 450 nm was measured using a spectrophotometer (Promega, Madison, WI, United States).

### Anti-Influenza Viral Assay

The influenza A/PR8-GFP virus (PR8-GFP IAV) was incubated with EBL or sulforaphane at indicated concentrations for 1 h at 4°C, and the mixtures were added to RAW 264.7 cells for 2 h at 37°C. After washing with PBS, the cells were further incubated until GFP expression. The level of GFP expression was detected using fluorescence microscopy or fluorescence-activated cell sorting (FACS) analysis.

### Immunofluorescence Staining

RAW 264.7 cells were seeded and incubated with a mixture of PR8-GFP IAV and 200 μg/ml of EBL for 2 h at 37°C. After further 24 h incubation, the cells were fixed with cold absolute methanol for 10 min and 4% paraformaldehyde for 10 min. The cells were subjected to blocking with 1% BSA-PBS for 30 min and incubated with antibodies (GeneTex, Irvine, CA, United States) against influenza viral M2, NP, and HA proteins for 1 h at room temperature. After washing with PBS containing 0.05% Tween 20 (PBST), the cells were incubated with Alexa Fluor 594-tagged secondary antibody in PBST for 1 h in the dark and followed by incubation with Hoechst 33342 for 5 min. The images of red viral proteins and blue nuclei were visualized under fluorescence microscopy.

### Fluorescence-Activated Cell Sorting Analysis

EBL at 0, 10, 100, or 200 μg/ml was incubated with PR8-GFP IAV for 1 h at 4°C, and the mixtures were added and incubated in RAW 264.7 cells for 24 h at 37°C. The cells were harvested, washed with PBS, and fixed with 4% paraformaldehyde. The cells were resuspended in PBS, and GFP expression was analyzed using a CytoPLEX flow cell counter (Beckman Coulter Inc., Pasadena, CA, United States).

### Hemagglutination Assay

The influenza A/PR/8/34 (H1N1) virus and 0, 10, 100, 200, and 400 μg/ml of EBL were cotreated for 1 h at 4°C, and each mixture was added to RAW 264.7 cells for 2 h at 37°C. After washing with PBS, the cells were further incubated for 24 h. The supernatant was used for hemagglutination assay. Briefly, each supernatant was serially two-fold diluted and added to a round-bottomed 96-well plate. The blank medium was used as a negative control. Each sample was mixed with an equal volume of 1% chicken RBCs (Innovative Research, Inc., Southfield, MI, United States) in PBS and incubated for 1 h at room temperature. RBCs in negative control exhibited agglutination, and RBCs in the virus-infected well were lysed without agglutination. HA titers were calculated as HA unit/100 µl in comparison with values of the control treatment. HA units were determined as the point at which hemolysis occurs.

### Neuraminidase Inhibition Assay

Neuraminidase (NA) inhibition assay was performed using the NA-Fluor influenza Neuraminidase Assay Kit (Life Technologies, Carlsbad, CA, United States) performed according to the manufacturer’s instructions. EBL was serially diluted from 1000 μg/ml to 1 μg/ml in 96-well black plates. As a positive control, oseltamivir carboxylate (AOBIOUS INC, MA, United States), a specific NA inhibitor, was used from 10 to 0.01 μM. Influenza A/PR/8/34 virus was added to each well and incubated for 30 min at 37°C. The mixtures were incubated with 200 µM NA-Fluor Substrate for 1 h at 37°C and terminated by NA-Fluor stop solution. The reaction was monitored using a fluorescence spectrometer (Promega, Madison, WI, United States), with an excitation wavelength of 365 nm and an emission wavelength of 445 nm.

### Time of Addition Assay

Time-of-addition assay was performed on binding, entry, and virucidal stage upon IAV infection. For the attachment stage, RAW 264.7 cells were infected with PR8-GFP IAV and 200 μg/ml EBL at 4°C for 30 min. After washing, the cells were incubated at 37°C for 24 h. For the entry stage, the cells were infected with PR8-GFP IAV at 4°C for 30 min and followed by the treatment of EBL at 37°C for 30 min. After washing, the cells were incubated at 37°C for 24 h. For the virucidal stage, PR8-GFP IAV and EBL were incubated at 4°C for 30 min. The mixtures were added to the cells and incubated at 37°C for 30 min. After washing, the cells were incubated at 37°C for 24 h. The brightfield and fluorescence images of cells were captured under fluorescence microscopy with ×200 magnification. The cells were fixed and analyzed by FACS.

## Data Availability

The raw data supporting the conclusion of this article will be made available by the authors, without undue reservation.
