# Supplementary material for: Broccoli Leaves Attenuate Influenza A Virus Infection by Interfering With Hemagglutinin and Inhibiting Viral Attachment
Source: Front Pharmacol. 2022 Jun 30;13:899181. doi: 10.3389/fphar.2022.899181 (PMC9280179; doi:10.3389/fphar.2022.899181)
Supplement: Supplementary file 1 [file Image1.pdf]

Supplementary material

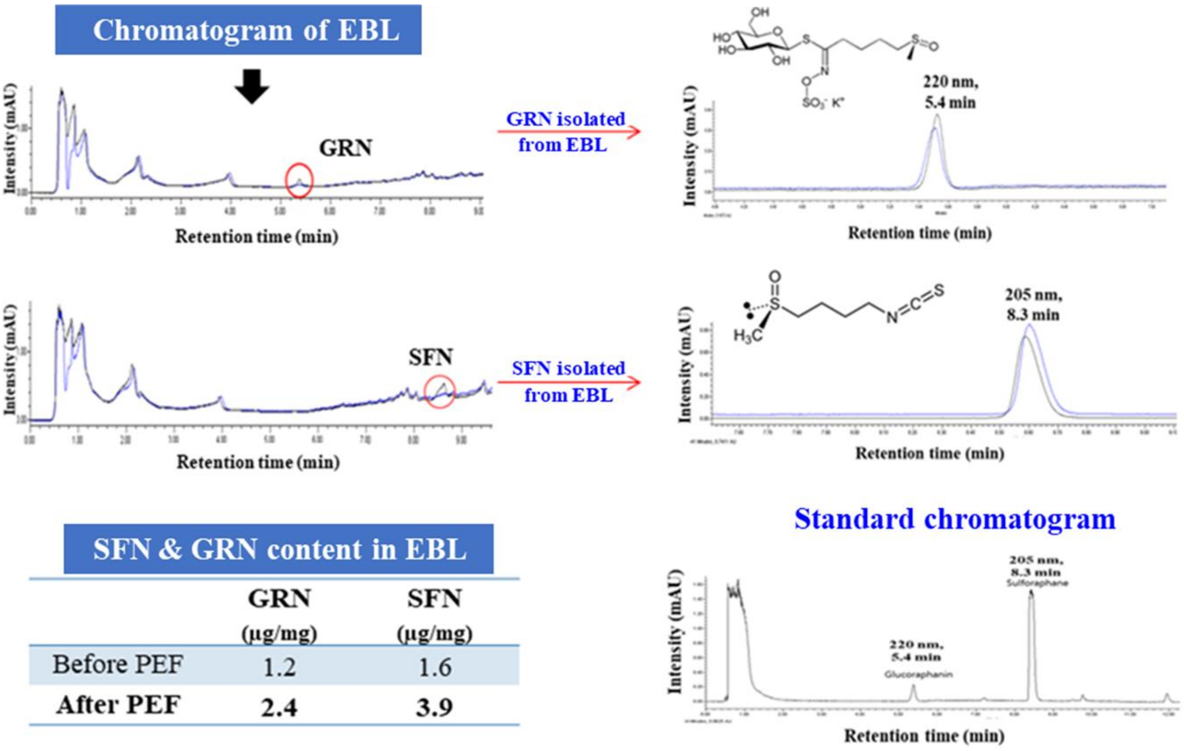

	GRN (μg/mg)	SFN (μg/mg)
Before PEF	1.2	1.6
**After PEF**	**2.4**	**3.9**

Figure S1. UPLC analysis of EBL.

The UPLC analysis was performed to determine the presence of SFN and GRN in EBL. The retention time of SFN and GRN, isolated from the broccoli leaves were matched with the standard chromatogram. The concentrations of SFN and GRN in the freeze-dried broccoli powder were 3.99 ± 0.09 μg/mg and 2.46 ± 0.2 μg/mg respectively. The results confirmed that EBL prepared by PEF treatment contained SFN and GRN.
